# Supplementary material for: Extracellular Nicotinamide Phosphoribosyltransferase Is a Therapeutic Target in Experimental Necrotizing Enterocolitis
Source: Biomedicines. 2024 Apr 28;12(5):970. doi: 10.3390/biomedicines12050970 (PMC11118767; doi:10.3390/biomedicines12050970)
Supplement: Supplementary file 1 [file biomedicines-12-00970-s001.zip › biomedicines-2901977-supplementary.pdf]

Supplemental Figures

Supplementary Materials: The following supporting information can be downloaded at: www.mdpi.com/xxx/s1, Figure S1. Principal component analysis of genes/pathways associated with innate immune responses, cytotoxicity, inflammation, NK-mediated immunity, and autoimmunity showed excellent separation (A) and impressing differential gene expression (B) between NEC (n = 3) vs NEC + mAb (n = 3); Figure S2. RNA sequencing and differential gene expression analysis of NEC (n = 3) and NEC + mAb groups (n = 3); Figure S3. GSEA analysis of the RNAseq dataset against the GSEA gene set for Hallmark of TGF beta signaling profile of running Enrichment Score (ES) of 0.5, Normalized Enrichment Score (NES) of 1.84, Nominal p value<0.001, and FDR q- value of 0.000 (Pathway on the y-axis indicates positive correlation with NEC treated with eNAMPT mAb compared with NEC saline-treated samples.

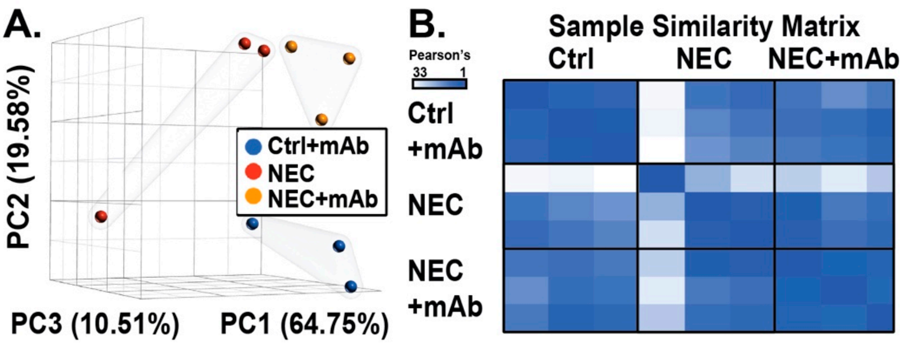

Supplementary Figure S1. Principal component analysis of genes/pathways associated with innate immune responses, cytotoxicity, inflammation, NK-mediated immunity, and autoimmunity showed excellent separation (A) and impressing differential gene expression (B) between NEC (n = 3) vs NEC + mAb (n = 3).

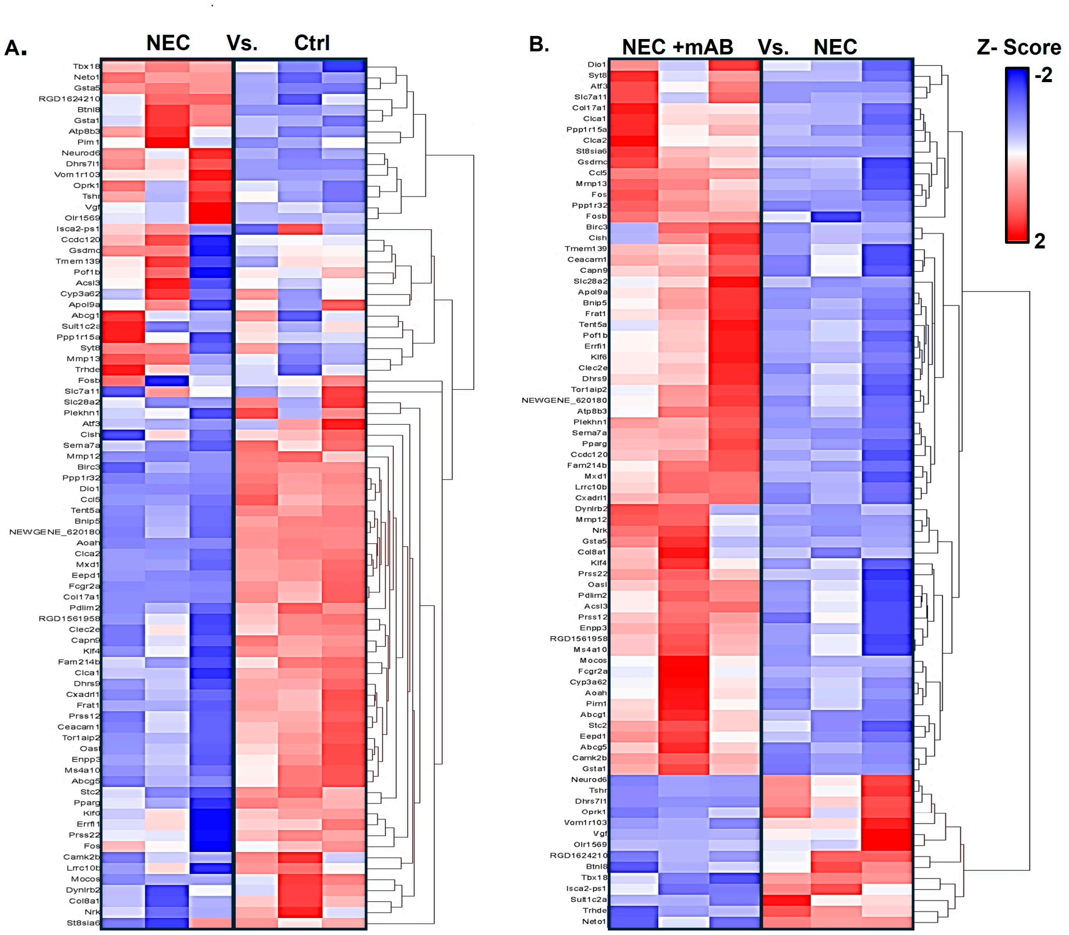

Supplementary Figure S2. RNA sequencing and differential gene expression analysis

of NEC (n = 3) and NEC + mAb groups (n = 3).

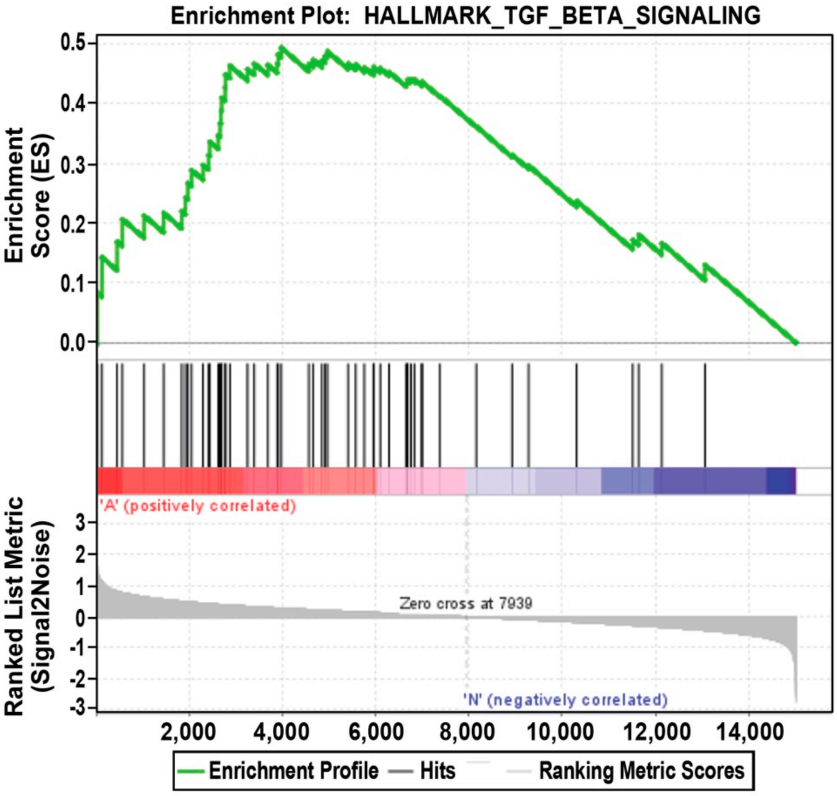

Supplementary Figure S3. GSEA analysis of the RNAseq dataset against the GSEA gene set for Hallmark of TGF beta signaling profile of running Enrichment Score (ES) of 0.5, Normalized Enrichment Score (NES) of 1.84, Nominal p value<0.001, and FDR q- value of 0.000 (Pathway on the y-axis indicates positive correlation with NEC treated with eNAMPT mAb compared with NEC saline-treated samples.
